# Some psychometric properties of the Chinese version of the Modified Dental Anxiety Scale with cross validation

**DOI:** 10.1186/1477-7525-6-22

**Published:** 2008-03-25

**Authors:** Siyang Yuan, Ruth Freeman, Satu Lahti, Ffion Lloyd-Williams, Gerry Humphris

**Affiliations:** 1 Dental Health Research Unit, Mackenzie Building, Ninewells Hospital, University of Dundee, UK; 2 Department of Community Dentistry, University of Oulu, Finland; 3 Oral and Maxillo-facial Department, Oulu University Hospital, Oulu, Finland; 4 Department of Public Health, University of Liverpool, UK; 5 Health Psychology, Bute Medical School, University of St-Andrews, UK

## Abstract

**Objective:**

To assess the factorial structure and construct validity for the Chinese version of the Modified Dental Anxiety Scale (MDAS).

**Materials and methods:**

A cross-sectional survey was conducted in March 2006 from adults in the Beijing area. The questionnaire consisted of sections to assess for participants' demographic profile and dental attendance patterns, the Chinese MDAS and the anxiety items from the Hospital Anxiety and Depression Scale (HADS). The analysis was conducted in two stages using confirmatory factor analysis and structural equation modelling. Cross validation was tested with a North West of England comparison sample.

**Results:**

783 questionnaires were successfully completed from Beijing, 468 from England. The Chinese MDAS consisted of two factors: anticipatory dental anxiety (ADA) and treatment dental anxiety (TDA). Internal consistency coefficients (tau non-equivalent) were 0.74 and 0.86 respectively. Measurement properties were virtually identical for male and female respondents. Relationships of the Chinese MDAS with gender, age and dental attendance supported predictions. Significant structural parameters between the two sub-scales (negative affectivity and autonomic anxiety) of the HADS anxiety items and the two newly identified factors of the MDAS were confirmed and duplicated in the comparison sample.

**Conclusion:**

The Chinese version of the MDAS has good psychometric properties and has the ability to assess, briefly, overall dental anxiety and two correlated but distinct aspects.

## Background

The assessment of dental anxiety is becoming increasingly relevant with the stronger emphasis on evidence based methods for improving patient oral health care [1,2]. In particular, recording self-reported dental anxiety in those patients who report psychological difficulties in receiving dental treatment enables planners of dental services to make informed decisions about suitable interventions [1,3]. This is especially important in countries like China that are experiencing rapid economic development. China's health services are receiving close attention as its population is drawn into utilizing a mix of traditional and western influenced primary care provision. Dental services are expanding and little evidence is currently available on the factors responsible for uptake, of which dental anxiety is a likely candidate for explaining utilisation.

Issues that govern the choice and the use of dental anxiety measures in clinical practice and epidemiological surveys are: number of question items, complexity, validity and useability [4]. There are a number of self-reported measures of dental anxiety that vary in length, theoretical background and psychometric evidence [5]. Some scales are available in a variety of languages e.g. [6-8]. A popular measure of dental anxiety was the four item Corah's dental anxiety scale [9], however this scale omits assessing respondents' views to dental anaesthesia and has a complex answering scheme. The 5 item modified dental anxiety scale (MDAS) was constructed to satisfy both problems by introducing a new item about local anaesthesia and simplifying the response format [10]. Conversion tables are available [11]. A clinical cut-off score of 19 and above has been determined to identify highly dentally anxious individuals who require specialist care (e.g. behavioural management and/or anaesthesia) [10]. A diagnostic classification for dental phobia has been devised based upon international criteria [12].

There are issues of usability that concern, first, how long the questionnaire takes to complete and, second the effect of instrumentation. An example of the first issue is the 36 item questionnaire (Dental Anxiety Inventory, DAI) designed to assess 3 'facets' of dental anxiety [13]. Although highly reliable it was found to be impractical in clinical settings because of the relatively long completion time [14]. A shorter 8 item version has been devised [15]. The second issue of instrumentation has received little interest hitherto. There is some evidence that dental personnel are concerned about the possibility of raising dental anxiety by inviting patients to report their feelings associated with a dental visit [16]. The design and subsequent development work with the Modified Dental Anxiety Scale has attempted to address this concern. The MDAS is brief and requires just 2–3 minutes to complete [10]. Moreover, and crucially, the scale does not raise anxiety in respondents, regardless of their initial level of dental anxiety [17,18] and rather than be detrimental its completion can be beneficial to patients when incorporated into managed care procedures within a practice setting [19].

The MDAS has been validated in the UK [10,20,21] and a number of other countries with native translations: Finnish, Arabic, Hindi [20] Turkish [22,23], Norwegian [24], German, Portuguese and Rumanian [25]. A previous report has demonstrated the validity of the Mandarin version of the short DAI [14], however the scale consists of 8 items and for clinical purposes, and inclusion in large epidemiological surveys, the shorter MDAS may be considered more suitable. The current study was motivated to develop the Chinese version of the MDAS that would be reliable and valid. Reliability was to be tested employing methods that reduce the number of assumptions used by traditional tests (explained below), and the scale's construct validity was checked by reference to the predicted relationships of the scale with a number of demographic and behavioural variables, and some tests of the structural relationships with other related constructs including general anxiety.

To date most dental anxiety scales have received limited attention to their theoretical underpinnings. Dental anxiety is not unitary and has been typically conceived under three connected approaches: behavioral, cognitive and physiological. Self-report methods primarily assess the cognitive component which can be split into at least two valid constructs [26] 'exogenous and endogenous, with respect to the source of their anxiety'[27]. The former describes dental anxiety as a conditioned response whereas the latter refers to a constitutional vulnerability to anxiety disorders. A dental anxiety measure that could feasibly capture some aspects of these two constructs would improve our understanding and hence treatment planning.

The MDAS although designed as a general screening instrument of dental anxiety requires further investigation to ascertain whether it is unitary. On inspection of the item content it can be hypothesised that the first two items constitute anticipatory dental anxiety (ADA) whereas the final three items tap emotions raised by the thought of having various dental treatments, that could be termed treatment dental anxiety (TDA). The separation of the scale into these two components may assist researchers and clinicians in understanding patient reaction to a dental visit. This proposed two factor model can be tested by adopting confirmatory factor analysis. This approach is particularly helpful for the researcher when a clear measurement structure is proposed [28,29]. Various indexes of fit can be inspected to assess the proximity of the raw item responses to the hypothesised model [30]. Not only can the measurement model be tested with the total sample collected but also comparisons can be made across important groups within the sample. It was expected that the Chinese MDAS would show higher levels of dental anxiety with females than males supporting previous findings [31,23,32] and lending support to the construct validity of the scale. In addition, older people and regular dental attenders are known to be less dentally anxious than their younger and irregular dental attending counterparts, respectively [10]. These effects were predicted with the Chinese MDAS measure.

It is curious, that there is a high frequency of researchers demonstrating a sex difference in dental anxiety level, although no previous report has investigated the structure of responses to self report dental anxiety measures across gender. To maintain clarity of interpretation of the total scale score it would be an important feature of an assessment to show consistency of the measurement structure across gender.

The term dental anxiety was first conceptualised as a theoretical construct to understand the relationship between previous and frightening dental treatment experiences with the affect experienced when attending for dental treatment [33]. This allowed dental anxiety to be formulated in terms of anticipatory anxiety to explain how anxious patients relived the original frightening experience when attending the dentist for treatment in the present [34,35]. Furthermore, it was postulated that dental anxiety was related to an individual's general anxiety [36,37]. Previous work with general anxiety scales, such as the HADS (from a large non-clinical sample: n = 2547) has shown that the anxiety subscale consists of two constructs: namely, negative affectivity (NA, items 1,5,7) and autonomic anxiety (AA, items 3,9,13) [38]. Autonomic anxiety (AA) refers to high levels of autonomic arousal characterised by somatic symptoms such as shakiness, trembling and feelings of panic [39] whereas negative affectivity (NA) has been described as a 'temperamental sensitivity to negative stimuli' [40] or general distress [41]. We posited that the AA subscale would be strongly associated with the anticipatory dental anxiety (ADA) items of the MDAS as individuals who tend to be 'physiological reactors' [42] will score highly on items that indicate imminent future exposure to the dental situation. Whereas individuals who suffer high levels of negative affectivity (NA) may be particularly likely to respond negatively to a wide variety of specific dental procedures (i.e. indiscriminate response across situations [41] page 466) and therefore accumulate high levels of Treatment Dental Anxiety (TDA). Such a pattern of relationships, if found in observed data, would help to confirm the construct validity of the MDAS. The generalisability of this structural model would be reflected if these relationships were found in more than one sample. We considered performing a strict test of this model with two samples from very different cultures (Chinese and English). If equivalence of relationships between the two cultural groups were found then this would aid our understanding of how dental anxiety was conceived by the two groups of respondents as well as support the validity of the measure. A similar approach has been reported previously, but without employing methodology to formally test for equivalence [43]. There is some evidence that Chinese people remember past events in a different way to people from western cultures [44]. Caucasians tend to reflect on single significant personal incidents, whereas Chinese will concentrate on situations that have greater societal importance and reduce the emphasis on individual past experiences [44].

Hence the overall aim of the present study was to assess the factorial structure and construct validity for the Chinese version of the Modified Dental Anxiety Scale (MDAS). The specific objectives were to:

1. To test the factorial structure of the Chinese version of the MDAS and confirm its integrity across an important demographic categorisation, namely: gender.

2 To investigate further the psychometric properties of this version of the MDAS by assessing first its reliability, second its construct validity through predicted relationships with demographic, behavioral and psychological constructs and thirdly, the consistency of the relationships of general and dental anxiety across cultures (Chinese and North-west of England).

## Method

### The sample

Ethical approval was obtained from Beijing Hospital, Ethical Committee. Data was collected from March to April 2006. A convenience sample aged between 16 and 80 years was recruited from urban areas of four districts in Beijing, namely Dong Cheng, Hai Dian, Feng Tai and Fang Shan. The survey was completed in the work setting and involved three large energy supply and generating companies (greater than 3000 employees) which were state run and a small number of moderate to small size non-manufacturing firms consisting of 50 to 100 employees. Data was collected by one of the authors (SY) with four trained volunteer interviewers in the staff common rooms. Prior to the process of data collection, these volunteer interviewers received training to ensure they expressed neutral attitudes towards participants and their consistency of introducing the research, soliciting consent from participants and giving instructions on how to complete the questionnaire.

The North west of England sample was obtained from patients attending their general dental service practitioner in the waiting rooms of two practices (urban and rural setting) in a regional funded study to assess practitioners' recognition of mental health problems in primary care.

### The questionnaires

The questionnaire consisted of the participants' demographic profile, dental attendance patterns and the Chinese versions of the MDAS and the HADS. The MDAS asks respondents to indicate their emotional reaction to a dental visit, when in the waiting room, drilling, scaling and local anaesthetic injection. The MDAS uses a simple rating scale with 5 possible responses to each question. The responses range from 'not anxious' (scoring 1) to 'extremely anxious' (scoring 5). Reliability of the English language version of the MDAS is good (internal consistency = 0.89; test-retest = 0.82) [10,20]. The first author (SY) produced a Chinese language version (standard Mandarin) and back translated the scale. In addition, four Chinese residents who also spoke English and were naïve about the aims and processes of this research, gave independent assessment of the translations. A Chinese language expert back translated the questionnaire into English and compared their version with the first author. Any differences were resolved by consensus. Translation of the questionnaire was also tested in a pilot sample of 10 Chinese adults to ensure that every question of hospital based anxiety questionnaire was fully understood for people with different literacy level.

The Chinese version of the HADS anxiety subscale was used [45]. This was composed of seven items each with a 4 category rating answering scheme. Scores were derived by summing items together. This recent report confirmed the factorial structure of the HADS using the Dunbar model which we have applied in this paper [38], although a single factor also achieved a similar fit. The HADS is a widely used measure to assess psychological distress and has been designed to prevent the measure from tapping emotional responses to acute symptoms such as pain [46]. It has been translated into many languages, applied to a variety of settings and has a high level of acceptability.

The North west England sample completeded the English versions of the MDAS and HADS questionnaires plus items on demographics and dental attendance behaviour.

### Administration of the questionnaire

Both samples in China and England were approached by the researchers with an information sheet, consent obtained and issued with the questionnaire. No direction was provided to prevent response bias. Questionnaires were checked for completeness on return.

### Statistical analysis

The data were entered into SPSSv12 and imported into AMOSv6 [47]. We followed two major stages of analysis as recommended [48] coincident with our two objectives. The first stage consisted of confirmatory factor analysis (CFA) to demonstrate the hypothesised factorial structure of the MDAS and perform an omnibus test to ascertain parameter equivalence across gender to satisfy the first objective [28]. The second objective not only required some group comparisons using t-tests and fixed factor ANOVAs [49], but also the second major analytical stage of structural equation modelling (SEM) to formally test the expected relationships between general anxiety and dental anxiety.

The SEM approach allows important benefits to the researcher as issues of measurement error and the logical investigation of *a priori *structures of hypothesised latent factors composed of manifest indicators can be inspected [29]. SEM supersedes the simple reporting of correlation coefficients which suffer from interpretational difficulties due to a mixture of both systematic and random measurement error. Hence SEM analyses will enable efficient testing of the factorial structure (Objective 1) and assist with providing further evidence for the construct validity of the scale (Objective 2) by testing the strength of the hypothesised relationships between the dental anxiety scale and the HADS. In the current investigation it enabled equality constraints to be positioned on the covariances, across Chinese and English respondents. This provided the opportunity to test for equivalence between these two groups. Such comparisons between relevant groups act as a preliminary stage in understanding cultural differences in general and dental anxiety relations.

Maximum likelihood was the preferred method for estimating all parameters in the CFA and SEM analyses, consistent with convention especially with large sample sizes. However asymptotic distribution free estimation was also applied to check for discrepancy in overall results that might result from deviation of variables from multivariate normal distribution. A number of fit indices were employed to provide an overall assessment of fit of the raw data to the specified model (RMSEA, GFI, CFI and NFI) and also to compare alternative models (chi square difference test) [50].

## Results

### The samples

791 participants were approached in the Beijing area to participate in the study, 8 people refused to take part due to time constraints or inconvenience. Complete data were available from 783 respondents. The response rate was 99%. Demographic and typical attendance history data are presented (Table 1). The data set from the North-west of England comprised 468 respondents of whom 58.3% (273/468) were female, 19% aged 16–30 years, 49% aged 31–50 years and 31% aged 51 years or above. Sixty-two percent self-reported that they attended at least every 6 months, 37% only when in trouble and 1% had never attended previously.

**Table 1:** Demographics and dental status and care habits for Beijing sample

	**N**	**%**
**Gender**		
Male	358	45.7
Female	425	54.3
		
**Age**		
16–17	45	5.7
18–30	359	45.8
31–50	288	36.8
50–65	66	8.4
66–80	25	3.2
		
**Education**		
Junior High School	99	12.6
Senior High School	202	25.8
Higher Education	482	61.6
		
**Occupation**		
Farmers	57	7.3
Semi-Skilled	84	10.7
Skilled	240	30.7
Managerial	81	10.3
Professional	81	10.3
Student	205	26.2
Retired	28	3.6
Unemployed	7	.9
		
**Annual Income (RMB)**		
Under 20 K	443	56.6
20 K–80 K	296	37.8
Above 80 K	44	5.6
		
**Visiting the Dentist**		
Regular check up	82	10.5
Only when a problem	531	67.8
Never see a dentist	170	21.7
		
**Denture wearing**		
Complete denture	16	2.0
Partial removable denture	68	8.7
No denture, have own teeth	623	79.6
No denture, no teeth	76	9.7

Simply summing the 5 MDAS items together (range 5 to 25) and adopting an uncritical cut-off of 19, [10] it was found that 8.7% of the Chinese sample and 8.3% of the English sample may have high dental anxiety.

### Factorial structure

The Chinese MDAS data were subjected to confirmatory factor analysis (CFA), to test initially the unidimensional model, that is, all items loading onto a single latent variable (Model A). The correlation matrix and associated summary statistics are presented in the Table 2. The analysis demonstrated moderate fit (Table 3). Inspection of the modification indices (values greater than 25 was used as a criterion) demonstrated that there was some localised 'strain' (i.e. poor fit) in the model as specified [28]. This was signalled by evidence of a significant correlation between the two residual errors for the first two questions of the scale (namely 'mdas1' and 'mdas2'). The question content of these 2 items focused on the anticipation of anxiety before entering the dental surgery, hence these items (as hypothesised) shared some overlap. Hence the error covariance between these 2 items was allowed to correlate. The fit of the resulting model was improved considerably (Model B, Table 3) as shown by the substantial reduction in chi-square value with a single degree of freedom (the chi-square difference).

**Table 2:** Means, SDs and correlations of Chinese sample's dental anxiety (MDAS) and general anxiety (HADS)

	Item	mean	SD	1	2	3	4	5	6	7	8	9	10	11
1	mdas1	1.83	0.99	1										
2	mdas2	1.99	0.99	0.695	1									
3	mdas3	2.89	1.21	0.557	0.595	1								
4	mdas4	2.47	1.19	0.476	0.586	0.674	1							
5	mdas5	2.82	1.27	0.430	0.499	0.674	0.673	1						
6	h1	1.07	0.78	0.150	0.205	0.200	0.227	0.166	1					
7	h3	0.81	0.82	0.163	0.233	0.183	0.254	0.221	0.361	1				
8	h5	0.83	0.78	0.142	0.170	0.197	0.173	0.176	0.357	0.456	1			
9	h9	0.75	0.71	0.093	0.161	0.115	0.193	0.154	0.225	0.334	0.324	1		
10	h13	0.80	0.67	0.073	0.151	0.149	0.158	0.144	0.256	0.393	0.386	0.439	1	
11	h7	1.36	0.88	0.111	0.146	0.188	0.189	0.164	0.303	0.213	0.309	0.235	0.186	1

n = 783, all correlations significant p < .001

**Table 3:** Summary statistics of overall model fit for the conventional single factor version of the Chinese version of the MDAS

	χ^2^	*df*	χ^2 diff^	Δ*df*	RMSEA	GFI	CFI	NFI
Model A	206.2	5			.227	.902	.902	.901
Model B^†^	33.9	4	172.3*	1	.098	.983	.985	.984

Notes: χ^2 diff ^(χ^2 ^difference); root mean square error of approximation (RMSEA); goodness of fit index (GFI); comparative fit index (CFI); normative fit index (NFI);

^† ^as Model A but with correlated residual from 'mdas1' and 'mdas2';

* p < 0.0001.

**Figure 1:**
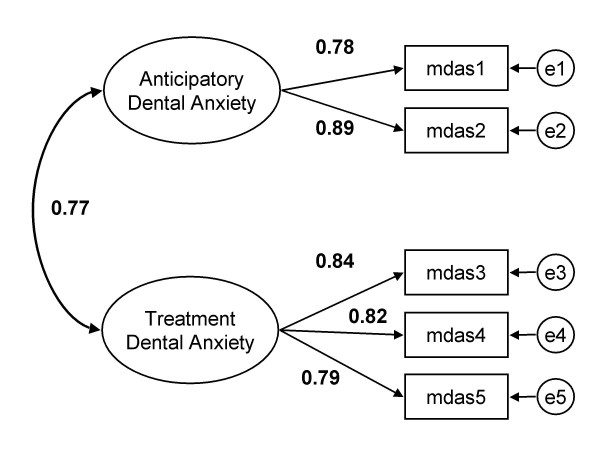
Measurement model of the two factor version of the MDAS with standardized parameter estimates.

On the strength of the positive evidence of overlap in item content of the first two MDAS questions the two factor model was specified in accordance with prediction (Figure 1). Items 1 and 2 comprised the anticipatory dental anxiety subscale (ADA). Items 3 to 5 described the proposed treatment procedure dental anxiety subscale (TDA). The two subscales were allowed to covary and all measurement error was assumed to be unsystematic, that is with no correlated errors specified. This model by definition gave an identical fit to Model B. This 2 factor model was invariant across gender, as tested by three increasingly stringent stages: (i) factor loadings; (ii) covariance between the two factors; and (iii) the error variances. These parameters for each element type (i–iii) were constrained in turn across gender to be equal and compared with the identical but unconstrained models. Results of these analyses (available on request from authors) showed equivalence at each step respectively (i) *p *> .7, (ii) *p *> .6, (iii) *p *= .07.

### Reliability

Cronbach's alpha, specifies that all of the items contribute equally to the underlying latent factor, a position that is often unsustainable [51]. Hence we calculated the reliability coefficients from the CFA results using the preferred method that does not assume Tau equivalence [28]. The two factor dental anxiety model from the MDAS was internally consistent as shown by the unbiased reliability coefficients 0.74 and 0.86 for the anticipatory and dental treatment factors respectively. Calculation of the more traditional Cronbach alphas (ADA = 0.82 and TDA = 0.86 respectively) supported our concern as the item covariances on the anticipatory items were far from equal (0.69 and 0.61). The treatment dental anxiety items exhibited less diversity (1.03, 1.00, 1.04) and hence there was little discrepancy in coefficients. These results were confirmed when models constraining the factor loadings to be equal (thereby *imposing *Tau equivalence) were run for each factor and compared to their counterpart models which were unconstrained. The chi-square difference was insignificant for the TDA factor (χ^2 ^= 0.44, df = 1, *p *= 0.51) and significant for the ADA factor (χ^2 ^= 7.58, df = 1, *p *= 0.006) as the observation of the covariances suggested.

### Construct Validity

The variance of dental anxiety as assessed by the Chinese MDAS was analysed across age, gender and self-reported dental visiting.

#### 1. Age

The older age group (greater than 50 years) had a significantly lower mean score for dental anxiety compared with younger age groups (those aged between 16 and 50 years). The mean (95%CI) MDAS values for the three age groups were as follows: 16–30 years = 12.22, (11.77, 12.69); 31–50 years = 12.04, (11.50, 12.57); 50+ years = 10.86 (9.91, 11.81), *F *= 3.24, df = 2, 782, *p *= .04.

#### 2. Gender

Women had significantly higher mean scores (95%CIs) for dental anxiety compared with men: 10.92, (10.45, 11.39) vs 12.90 (12.47, 13.33) (*t *= 6.08: df = 781 *p *< 0.001).

#### 3. Dental attendance pattern

Participants who attended the dentist for a regular check up had significantly lower mean scores that those who attended only when experiencing a problem: regular check up = 11.17, (10.17, 12.17); only when in trouble = 12.28, (11.89, 12.68); never visit = 11.48, (10.79, 12.18) (*F *= 3.40, df = 2, 782, *p *= .03).

#### 4. Relationship with anticipatory and autonomic anxiety

**Figure 2:**
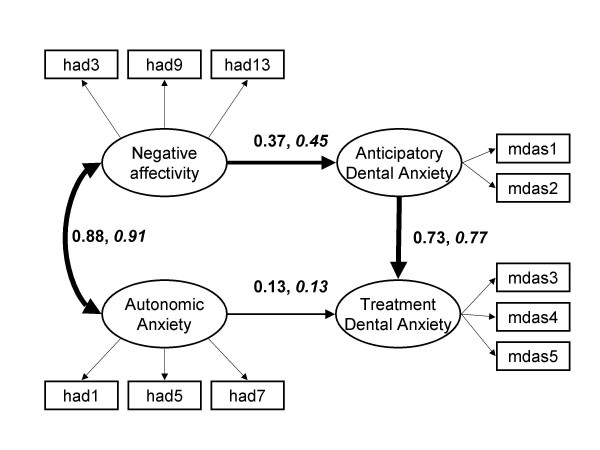
Structural model of the relation between negative affectivity, autonomic anxiety and the two factor version of the MDAS including standardised coefficients: Beijing and North-west England (italics). Wider arrows denote greater strength of relationship. Error terms omitted to simplify diagram.

The hypothesised structural model was evaluated with the Chinese data as specified in Figure 2. Standardised parameter estimates are shown. The correlation matrix is presented in Table 2. Of particular interest was the strength of the relationships between the anxiety latent factors (Negative Affectivity NA and Autonomic Anxiety AA) with the 2 dental anxiety latent factors (ADA and TDA). The results of the model fitting are summarised in Table 4.

**Table 4:** Summary statistics of overall fit for the hypothesized Model (i) with additional paths fitted as indicated by Models ii and iii

	χ^2^	*df*	χ^2 diff^	Δ*df*	RMSEA	GFI	CFI	NFI
Model i NA → TDA, AA → ADA, ADA → DTA, NA ↔ AA	98.44	40			.056	.964	.979	.966
Model ii As Model i plus NA → ADA	98.29	39	0.15^ns^	1	.057	.983	.985	.984
Model iii As Model i plus AA → TDA	96.93	39	1.51^ns^	1	.057	.964	.980	.967

Notes: χ^2 ^difference (χ^2 diff^); root mean square error of approximation (RMSEA); goodness of fit index (GFI); comparative fit index (CFI); normative fit index (NFI); ns = non significant (*p *> .05).

Alternative models were also tested. Negative affectivity may influence not only ADA but also TDA. Hence the path NA → TDA was included (Model ii, Table 4) which resulted in a non-significant parameter estimate and little contribution to the overall fit. The further model of AA influencing directly ADA was also tested (i.e. path AA → ADA) (Model iii, Table 4). This path was also redundant.

Constraining the parameter estimates of all latent factor paths and the covariance (i.e. NA → TDA, AA → ADA, ADA → TDA, NA ↔ AA) to be equal across the two national samples (correlation matrices, means and SDs presented in Tables 2 and 5) showed no significant diminution of fit (omnibus test, *p *= .16). The paths NA → TDA and AA → ADA were significant in both samples (*p *< .001). However the strength of the AA → ADA appeared quantitatively larger as predicted from theory.

**Table 5:** Means, SDs and correlations of English sample's dental anxiety (MDAS) and general anxiety (HADS)

	Item	mean	SD	1	2	3	4	5	6	7	8	9	10	11
1	mdas1	1.89	1.07	1										
2	mdas2	1.91	1.07	0.881	1									
3	mdas3	2.51	1.24	0.716	0.705	1								
4	mdas4	1.59	0.98	0.551	0.578	0.599	1							
5	mdas5	2.52	1.24	0.630	0.658	0.774	0.507	1						
6	h1	1.06	0.69	0.339	0.375	0.322	0.225	0.363	1					
7	h3	0.98	0.98	0.341	0.343	0.318	0.245	0.286	0.414	1				
8	h5	1.01	0.85	0.268	0.302	0.292	0.183	0.313	0.524	0.508	1			
9	h9	0.88	0.63	0.300	0.312	0.307	0.243	0.300	0.442	0.501	0.493	1		
10	h13	0.79	0.78	0.340	0.338	0.330	0.262	0.295	0.435	0.503	0.547	0.556	1	
11	h7	0.87	0.67	0.294	0.313	0.300	0.312	0.193	0.480	0.372	0.433	0.390	0.435	1

n = 468, all correlations significant p < .001

Comparisons were made between the samples from Beijing and North-west of England using the MDAS total score and subscale data (Table 6). Univariate analysis of variance indicated that the Total MDAS scale scores showed an overall raised dental anxiety level in the Chinese sample compared with the English sample (*F *= 20.51, df = 1, 1271, *p *< .001) after controlling for age and sex. However similar analyses detected no difference between the groups on Anticipatory Dental Anxiety (ADA) (*F *= 0.08, df = 1, 1271, *p *= .77), whereas Treatment Dental Anxiety (TDA) was higher in Beijing compared with the North-west of England (*F *= 42.64, df = 1, 1271, *p *< .001).

**Table 6:** MDAS total and sub-scale scores (Anticipatory Dental Anxiety and Treatment Dental Anxiety) broken down by cross-cultural groups, namely: Beijing, China and the North-west of England. Means adjusted for age and sex.

	Group	Mean	95% Confidence Interval	
MDAS subscales			Lower	Upper
Anticipatory Dental Anxiety (ADA)	Beijing^1^	3.77	3.61	3.93
	NW England^2^	3.74	3.55	3.92
Treatment Dental Anxiety (TDA)	Beijing	7.91	7.65	8.17
	NW England	6.59	6.29	6.89
MDAS Total Score	Beijing	11.69	11.30	12.08
	NW England	10.32	9.88	10.77

1 N = 784

2 N = 489

The mean HADS anxiety sub-scale score was 6.63 (SD 3.43) and compares to the previous recent report in Xi'an province coronary heart disease (CHD) patients of 6.16 (SD 3.86) [45]. Thirty nine percent screened positive for anxiety compared to 32% of CHD patients using the recommended cut-off of 8 or over [46].

## Discussion

The overall aim of this investigation was to evaluate the psychometric properties (reliability and construct validity) of the Chinese version of the MDAS. Evidence was found to support a two factor structure for the Chinese MDAS. The two sub-scales identified were shown to be reliable.

In conducting this investigation we have demonstrated a number of new features in our understanding and testing of a dental anxiety self-report measure. First, whereas many previous reports provide reliability statistics for their dental anxiety measures, e.g. [9,20] this is the first study in the dental anxiety assessment field to report reliability coefficients relaxing the assumption of Tau equivalence. Where the range of factor loadings was narrow the disparity between Cronbach's alpha and internal consistency calculated with relaxed assumptions showed little difference. An unfortunate positive bias, however would have been present from maintaining the assumption of tau equivalence with the ADA scale.

Second, this study has revealed that the factorial structure of the Chinese MDAS can be viewed as two components, namely anticipatory and treatment related dental anxiety. The original MDAS was designed as a screen for use clinically in dental surgeries and also as a brief one-dimensional measure in epidemiological studies. There may be some merit in reporting the two component sub-scale scores as well as the overall total score in future studies as each subscale appears to demonstrate reasonable reliability and some validity as discussed further below. We accept the criticism of some authors who state that measures of dental anxiety that are restricted to a single dimension tend to minimise the complexity of the multifactorial phenomena that characterises the dental anxious individual [14,52]. In recognition of this researchers who wish to collect brief information about dental anxiety are able to test hypotheses that include aspects related to anticipation or to treatment. Furthermore the theoretical formulation and model testing supported the view that the ADA scale taps 'exogenous' whereas the TDA assesses 'endogenous' dental anxiety.

Third, this is the first investigation of a dental anxiety scale, namely the Chinese MDAS, which has determined the factorial structure to be equivalent across gender. Although some authors [7] commendably make comparisons with regard to the factorial structure and gender of dental anxiety assessments so that the data can be pooled, these comparisons are not formally tested but reliant on simple observation. The use of CFA enables formal testing of the factor loadings for each item being comparable across gender. Additional tests were performed that enabled statistical comparison of item error variances and the factor covariance to be identical across gender. The results demonstrated that the two factor model held well for both genders even though the levels of dental anxiety were significantly different as reflected in many previous reports. This has important clinical implications since males and females with low and high dental anxiety scores exhibit similar interpretation and patterns of responses to the questionnaire. Hence the MDAS can be used with confidence with patients presenting with varying degrees of dental anxiety.

Finally, this is the first study to demonstrate the structural equivalence of dental anxiety measures across cultures using SEM methodology. This a further example of relationships between constructs showing remarkable consistency across national groupings even though the mean levels of the variables under study may vary under normal circumstances considerably. Interestingly, a previous study employing SEM procedures has reported a non significant association of general anxiety with dental anxiety [53]. The strength of this Norwegian investigation was that it featured the assessment of anxiety using multiple measures. However, the work focused specifically on patients with *severe *dental anxiety and hence the range of variation in associating dental anxiety with other psychological measures would have been dramatically reduced. Hence this makes comparison of our data with Hakeberg's work somewhat tenuous.

In support of the construct validity of the Chinese version there was a number of expected relationships with gender, age and dental attendance. Although this set of results was somewhat gratifying in providing additional confidence in the ability of this dental anxiety assessment to reflect commonly reported effects, a further confirmation of the measurement properties of the scale was achieved with the derived pattern of parameters comprising the 'nosological net' of predictions resulting from theory about general anxiety phenomena and specific anxieties associated with the dental setting. A recent study (written in Chinese) with 3000 dental clinic patients in China demonstrated a significant positive correlation (r = 0.404) between trait anxiety and dental anxiety [54]. The measurement approach was restricted to broad constructs rather than breaking the constructs into meaningful sub-scales as adopted in this present study, however the overall effect of shared variance between general and a more situation specific anxiety was confirmed [54]. The earlier study by Schwarz and Birn comparing Danish and Chinese adults found that the ease of response from participants from both cultures may be explained by the items used in the dental anxiety assessment (a version of Corah's dental anxiety scale). They argued that the questions were 'very particular' and referred to practical situations that 'most people can relate to irrespective of culture' and duration since last dental visit [43].

Some evidence was found to suggest that the anticipatory dental anxiety factor may be relatively stable across the two national communities in the two widely varying cultures but that treatment-related anxiety is considerably different. These differences, found with the TDA scale, may be attributed to the limited dental treatment experience of one culture compared to the other. This interpretation may be premature as previous work using less sophisticated assessment approaches reached different conclusions [43]. It is of interest to speculate that the higher level of treatment dental anxiety in the Chinese sample may be explained by the finding that Chinese dentists tend to be reluctant to use local anaesthesia as drilling is considered to feel 'suan' or 'sourish' sensation rather than painful. Hence Chinese patients may experience more painful treatments and give greater treatment anxiety ratings [55]. Similar findings of lower utilisation of local anaesthesia were found with Taiwanese dentists compared to Caucasian Americans [56]. Comparative work of this nature across cultures provides ample opportunities for examining the issues of experience of dental treatment and the development and maintenance of dental anxiety.

Limitations of this study include a cautionary note on our adoption of directional paths between constructs. Where these have been employed they are illustrative and imply a possible influence, but further evidence in longitudinal and experimental studies is required. In addition, we recognise the difficulties of comparing data derived from very different communities and using separate sampling strategies. A number of authors stress caution in making comparisons between different populations [57,58]. From one perspective however, it may be argued that the similarities found across the 2 national samples were high regardless of the different composition of samples and adoption of the resident language of the participants. Further investigation is required to determine the clinical efficacy of using the Chinese MDAS as a two factor instrument to assess anticipatory and treatment dental anxiety, and to test for suitable clinical cut offs for clinic populations.

## Conclusion

The Chinese version of the MDAS has exhibited suitable psychometric properties for epidemiological and research study. The assessment is brief, providing low participant burden, to give an estimate of overall dental anxiety. It has the capacity to be presented, in addition, as two correlated but distinct constructs.

## Abbreviations

AA Autonomic Anxiety; ADA Anticipatory Dental Anxiety; CFA Confirmatory Factor Analysis; CFI Comparative Fit Index; CHD Coronary Heart Disease; GFI Goodness of Fit Index; HADS Hospital Anxiety and Depression Scale; MDAS Modified Dental Anxiety Scale; NA Negative Affectivity, NFI Normed Fit Index; RMSEA Root Mean Square Estimate of Approximation, SEM Structural Equation Models; TDA Treatment Dental Anxiety

## Competing interests

The author(s) declare that they have no competing interests.

## Authors' contributions

RF and GH conceived the study. GH participated in the design of the study, analysed the data and drafted the article. RF participated in the study design, contributed to the manuscript and coordinated the Chinese data collection. SY organized the Chinese data collection, trained the interviewers, prepared the data and commented on the various draft manuscripts. SL edited manuscript drafts. FLW organized and collected the North-west England sample, prepared data and provided initial results. All authors read and approved the final manuscript.
